# Supplementary material for: Heat adaptation of phage T7 under an extended genetic code
Source: Virus Evol. 2021 Dec 1;7(2):veab100. doi: 10.1093/ve/veab100 (PMC8923235; doi:10.1093/ve/veab100)
Supplement: veab100_Supp [file veab100_supp.zip › Sequencing_Analysis.zip!/Sequencing_Analysis/A2/evidence/RA_30.html]

BRESEQ :: Evidence


| Read alignment evidence... | | | | | | | | | | | |
| --- | --- | --- | --- | --- | --- | --- | --- | --- | --- | --- | --- |
|  | seq id | position | | ref | new | freq | score (cons/poly) | reads | annotation | genes | product |
| \* | NC\_001604.1 | 32,870 | 0 | G | T | 20.5% | 977.7 / 85.1 | 405 | intergenic (–/–) | *–/–* | –/– |
| Reads supporting (aligned to +/- strand):  **ref** base G (177/144);  **new** base T (44/39);  **total** (222/183) | | | | | | | | | | | |
| Fisher's exact test for biased strand distribution *p*-value = 8.05e-01 | | | | | | | | | | | |
| Kolmogorov-Smirnov test that lower quality scores support variant *p*-value = 6.97e-01 | | | | | | | | | | | |

Only 100 of 420 total aligned reads displayed.  

`GTCGCCACAAAGGTACTTACGTTCCTCACGTATATGACCGTCATGCCAAGGCGCTGATGATTCAACGCTACGGTGCCGAAGGTTTGCAGGAAGGGATTGCCCGCTCATGGATGAACAGCTACGTCTCCAGACCTGAGGTCAAGGCCAGAGTCGATGAGATGCTTAAGGAATTACACGGGGTGAAGGAAGTAACACCAGAGATGGTAGAGAAGTACGCTATGGATAAGGCTTATGGTATCTCCCACTCAGACCAGTTCACCAACAGTTCCATAATAGAAGAGAACATTGAGGGCTTAGTAGGTATCGAGAATAACTCATTCCTTGAGGCACGTAACTTGTTTGATTCGGACCTATCCATCACTATGCCAGACGGACAGCAATTCTCAGTGAATGACCTAAGGGACTTCGATATGTTCCGCATCATGCCAGCGTATGACCGCCGTGTCAATGGTGACATCGCCATCATGGGGTCTACTGGTAAAACCACTAAGGAACTTAAGGATGAGATTTTGGCTCTCAAAGCGAAAGCTGAGGGAGACGGTAAGAAGACTGGCGAGGTACATGCTTTAATGGATACCGTTAAGATTCTTACTGGTCGTGC  >  NC_001604.1/32570‑33170`  

                                                                                                                                                                                                                                                                                                            `|`                                                                                                                                                                                                                                                                                                              
               `cTTACGTTCCTCACGTATATGACCGTCATGCCAAGGCGCTGATGATTCAACACTACGGTGCCGAAGGTTTGCAGGAAGGGATTGCCCGCTCATGGATGAACAGCTACGTCTCCAGACCTGAGGTCAAGGCCAGAGTCGATGAGATGCTTAAGGAATTACACGGGGTGAAGGAAGTAACACC‑GAGATGGTAGAGAAGTACGCTATGGATAAGGCTTATGGTATCTCCCACTCAGACCAGTTCACCAACAGTTCCATAATAGAAGAGAACATCGAGGGCTTAGTAGTTATCGAGAATAACTCa                                                                                                                                                                                                                                                                                              >  1:521308/1‑301 (MQ=255)`  
                   `gatgtgtataagagacagccGTCATGCCAAGGCGCTGATGATTCAACGCTACGGTGCCGAAGGTTTGCAGGAAGGGATTGCCCGCTCATGGATGAACAGCTACGTCTCCAGACCTGAGGTCAAGGCCAGAGTCGATGAGATGCTTAAGGAATTACACGGGGTGAAGGAAGTAACACCAGAGATGGTAGAGAAGTACGCTATGGATAAGGCTTATGGTATCTCCCACTCAGACCAGTTCACCAACAGTTCCATAATAGAAGAGAACATTGAGGGCTTAGTAGGTATCGAGAATAACTCATTc                                                                                                                                                                                                                                                                                           <  1:201155/283‑1 (MQ=255)`  
                    `gTTCATCACGTATATGACCGTCATGCCAAGGCGCTGATGATTCAACGCTACGGTGCCGAAGGTTTGCAGGAAGGGATTGCCCGCTCATGGATGAACAGCTACGTCTCCAGACCTGAGGTCAAGGCCAGAGTCGATGAGATGCTTAAGGAATTACACGGGGTGAAGGAAGTAACACCAGAGATGGTAGAGAAGTACGCTATGGATAAGGCTTATGGTATCTCCCACTCAGACCAGTTCACCAACAGTTCCATAATAGAAGAGAACATTGAGGGCTTAGTAGGTATCGAGAATAACTCATTcc                                                                                                                                                                                                                                                                                          <  1:183300/301‑1 (MQ=255)`  
                       `ccTCACGTATATGACCGTCATGCCAAGGCGCTGATGATTCAACGCTACGGTGCCGAAGGTTTGCAGGAAGGGATTGCCCGCTCATGGATGAACAGCTACGTCTCCAGACCTGAGGTCAAGGCCAGAGTCGATGAGATGCTTAAGGAATTACACGGGGTGAAGGAAGTAACACCAGAGATTGTAGAGAAGTACGCTATGGATAAGGCTTATGGTATCTACCACTCAGACCAGTTCACCAACAGTTCCATAATAGAAGAGAACATTGAGGGCTTAGTAGGTATCGAGACTAACTAATTCCttt                                                                                                                                                                                                                                                                                       >  1:737701/1‑300 (MQ=255)`  
                            `cGTATATGACCGTCATGCCAAGGCGCTGATGATTCAACGCTACGGTGCCGAAGGTTTGCAGGAAGGGATTGCCCGCTCATGGATGAACAGCTACGTCTCCAGACCTGAGGTCAAGGCCAGAGTCGATGAGATGCTTAAGGAATTACACGGGGTGAAGGAAGTAACACCAGAGATGGTAGAGAAGTACGCTATGGATAAGGCTTATGGTATCTCCCACTCAGACCAGTTCACCAACAGTTCCATAATAGAAGAGAACATTGAGGGCTTAGTAGGTATCGAGAATAACTCATTCCCTgtctct                                                                                                                                                                                                                                                                                  >  1:490000/1‑296 (MQ=255)`  
                                `tatGACCGTCATGCCAAGGCGCTGATGATTCAACGCTACGGTGCCGAAGGTTTGCAGGAAGGGATTGCCCGCTCATGGATGAACAGCTACGTCTCCAGACCTGAGGTAAAGGCCAGAGTCGATGAGATGCTTAAGGAATTACACGGGGTGAAGGAAGTAACACCAGAGATGGTAGAGAAGTACGCTATGGATAAGGCTTATGGTATCTCCCACTCAGACCAGTTCACCAACAGTTCCATAATAGAAGAGAACATTGAGGGCTTAGTAGGTATCGAGAATAACTCATTCCTTGAGGCACGTa                                                                                                                                                                                                                                                                              >  1:395665/1‑301 (MQ=255)`  
                                      `cGTCATGCCAAGGCGCTGATGATTCAACGCTACGGTGCCGAAGGTTTGCAGGAAGGGATTGCCCGCTCATGGATGAACAGCTACGTCTCCAGACCTGAGGTCAAGGCCAGAGTCGATGAGATGCTTAAGGAATTACACGGGGTGAAGGAAGTAACACCAGAGATGGTAGAGAAGTACGCTATGGATAAGGCTTATGGTATCTCCCACTCAGACCAGTTCACCAACAGTTCCATAATAGAAGAGAACATTGAGGGCTTAGTAGGTATCGAGAATAACTCATTCCCTgtctcttatacacatt                                                                                                                                                                                                                                                                        >  1:528481/1‑286 (MQ=255)`  
                                             `ccAATGCGCTGATGATTCAACGCTACGGGGCCGAAGGTTCGCAGGAAGGGATTGCCCGCTCATGGATGAACAGCTACGTCTCCAGACCTGAGGTCAAGGCCAGAGTCGATGAGATGCTTAAGGAATTACACGGGGTGAAGGAAGTAACACCAGAGATGGTAGAGAAGTACGCTATGGATAAGGCTTATGGTATCTCCCACTCAGACCAGTTCACCAACAGTTCCATAATAGAAGAGAACATTGAGGGCTTAGTAGGTATCGAGAATAACTCATTCCTTGAGGCACGTAACTTGTTTGATTc                                                                                                                                                                                                                                                                 <  1:4713/301‑1 (MQ=255)`  
                                               `aaGGTGCTGATGATTCAACGCTACGGTGCCGAAGGTTTGCAGGAAGGGATTGCCCGCTCATGGATGAACAGCTACGTCTCCAGACCTGAGGTCAAGGCCAGAGTCGATGAGATGCTTAAGGAATTACACGGGGTGAAGGAAGTAACACCAGAGATGGTAGAGAAGTACGCTATGGATAAGGCTTATGGTATCTCCCACTCAGACCAGTTCACCAACAGTTCCATAATAGAAGAGAACATTGAGGGCTTAGTAGGTATCGAGAATAACTCATTCCTTGAGGCACGTCACTTGTTTGATTCgg                                                                                                                                                                                                                                                               >  1:298042/1‑301 (MQ=255)`  
                                                  `gcgcTGATGATTCAACGCTACGGTGCCGAAGGTTTGCAGGAAGGGATTGCCCGCTCATGGATGAACAGCTACGTCTCCAGACCTGAGGTCAAGGCCAGAGTCGATGAGATGCTTAAGGAATTACACGGGGTGAAGGAAGTAACACCAGAGATGGTAGAGAAGTACGCTATGGATAAGGCTTATGGTATCTCCCACTCAGACCAGTTAACCAACAGTTCCATAATAGAAGAGAACATTGAGGGCTTAGTAGGTATCGAGAATAACTCATTCCTTGAGGCACGTAACTTGTTTGATTcctggc                                                                                                                                                                                                                                                            >  1:190112/1‑296 (MQ=255)`  
                                                            `ttCAACGCTACGGTGCGGAAGGTTTGCAGGAAGGGATTGCCCGCTCATGGATGAACAGCTACGTCTCCAGACCTGAGGTCAAGGCCAGAGTCGATGAGATGCTGAATGAATTACACGGGGTGAATGAAGTAACACCATAGATGGTAGAGAAGTACGCTATGGATAAGGCTTATGGTATCTCCCACTCAGACCAGTTCACCAACAGTTCCATAATAGAAGAGAACATTGAGGGCTTAGTAGGTATCGAGAATAACTCATTCCTTGAGGCACGTAACTTGTTTGATTCGGACCTATCCATCAc                                                                                                                                                                                                                                                  <  1:787160/301‑1 (MQ=255)`  
                                                            `ttCAACGCTACGGTGCCGAAGGTTTGCAGGAAGGGATTGCCCGCTCATGGATGAACAGCTACGTCTCCAGACCTGAGGTCAAGGCCAGAGTCGATGAGATGCTTAAGGAATTACACGGGGTTAAGGAAGTAACACCAGAGATGGTAGAGAAGTACGCTATGGAGAAGGCTTATGGTATCTCCCACTCAGACCAGTTCACCAACAGTTCCATAATAGAAGAGAACATTGAGGGCTTAGTAGTTATCGAGAATAACTCATTCCTTGAGGCACGTAACTTGTTTGATTCGGACCTATCCATCAc                                                                                                                                                                                                                                                  <  1:329102/301‑1 (MQ=255)`  
                                                            `ttCAACGCTACGGTGCCGAAGGTTTGCAGGAAGGGATTGCCCGCTCATGGATGAACAGCTACGTCTCCAGACCTGAGGTCAAGGCCAGAGTCGATGAGATGCTTAAGGAATTACACGGGGTGAAGGAAGTAACACCAGAGATGGTAGAGAAGTACGCTATGGATAAGGCTTATGGTATCTCCCACTCAGACCAGTTCACCAACAGTTCCATAATAGAAGAGAACATTGAGGGCTTAGTAGGTATCGAGAATAACTCATTCCTTGAGGCACGTAACTTGTTTGATTCGGACCTATCCATCAc                                                                                                                                                                                                                                                  <  1:680403/301‑1 (MQ=255)`  
                                                            `ttCAACGCTACGGTGCCGAAGGTTTGCAGGAAGGGATTGCCCGCTCATGGATGAACAGCTACGTCTCCAGACCTGAGGTCAAGGCCAGAGTCGATGAGATGCTTAAGGAATTACACGGGGTGAAGGAAGTAACACCAGAGATGGTAGAGAAGTACGCTATGGATAAGGCTTATGGTATCTCCCACTCAGACCAGTTCACCAACAGTTCCATAATAGAAGAGAACATTGAGGGCTTAGTAGGTATCGAGAATAACTCATTCCTTGAGGAACGTAACTTGTTTGATTCGGACCTATCCATCAc                                                                                                                                                                                                                                                  <  1:590604/301‑1 (MQ=255)`  
                                                            `ttCAACGCTACGGTGCAGAAGGTTTGCAGGAAGGGATTGCCCGCTCATGGATGAACAGCTACGTCTCCAGACCTGAGGTCAAGGCCAGAGTCGATGAGATGCTTAAGGAATTACACGGGGTGAAGGAAGTAACACCAGAGATGGTAGAGAAGTACGCTATGGATAAGGCTTATGGTATCTCCCACTCAGACCAGTTCACCAACAGTTCCATAATAGAAGAGAACATTGAGGGCTTAGTAGTTATCGAGAATAACTCATTCCTTGAGGCACGTAACTTGTTTGATTCGGACCTATCCATCAc                                                                                                                                                                                                                                                  <  1:422431/301‑1 (MQ=255)`  
                                                            `ttCAAAGCTACGGTGCCGAAGGTTTGCAGGAAGGGATTGCCCGCTCATGGATGAACAGCTACGTCTCCAGACCTGAGGTCAAGGCCAGAGTCGATGAGATGCTTAAGGAATTAAACGGGGTGAAGGAAGTAACACCAGAGATGGTAGAGAAGTACGCTATGGATAAGGCTTATGGTATCTCCCACTCAGACCAGTTCACCAACAGTTCCATAATAGAAGAGAACATTGAGGGCTTAGTAGTTATCGAGAATAACTCATTCCTTGAGGCACGTAACTTGTTTGATTCGGACCTATCCATCAc                                                                                                                                                                                                                                                  <  1:417983/301‑1 (MQ=255)`  
                                                                  `gCTACGGTGCCGAAGGTTTGCAGGAAGGGATTGCCCGCTCATGGATGAACAGCTACGTCTCCATACCTGAGGTCAAGGCCAGAGTCGATGAGATGCTTAAGGAATTACACTGGGTGAAGGAAGTAACACCATAGATGGTAGAGAAGTACGCTATGGATAAGGCTTATGGTATCTCCCACTCAGACCAGTTCACCAACAGTTCCATAATAGAAGAGAACATTTAGGGCTTAGTAGGTATCGAGAATAACTCATTCCTTGAGGCACGTAACTTGTTTGATTCGGACCTATCCATCACTATGcc                                                                                                                                                                                                                                            <  1:33585/301‑1 (MQ=255)`  
                                                                        `gTGCCGAAGGTTTGCAGGAAGGGATTGCCCGCTCATGGATGAACAGCTACGTCTCCAGACCTGAGGTCAAGGCCAGAGTCGATGAGATGCTTAAGGAATTACACGGGGTGAAGGAAGTAACACCAGAGATGGTAGAGAAGTACGCTATGGATAAGGCTTATGGTATCTCCCACTCAGACCAGTTCACCAACAGTTCCATAATAGAAGAGAACATTGAGGGCTTAGTAGGTATCGATAATAACTCATTCCTTGAGGTACGTACCTTGTTTGATTCGGACCTATCCATCACTATGCCAGACgg                                                                                                                                                                                                                                      >  1:275834/1‑301 (MQ=255)`  
                                                                          `gCCGAAGGTTTGCAGGAAGGGATTGCCCGCTCATGGATGAACAGCTACGTCTCCAGACCTGAGGTCAAGGCCAGAGTCGATGAGATGCTTCAGGAATTACACGGGGTGAAGGAAGTAACACCAGAGATGGTAGAGAAGTACGCTATGGAAAAGGCTTATGGTATCTCCCACTCAGACCAGTTCACCAACAGTTCCATAATAGAAGAGAACATTCAGGGCTTAGCAGGTATCGAGAATAACTCATTCCTTGAGGCACGTAACTTGTTTGATTCGGACCTATCCATCACTATGCCAGCCGGAc                                                                                                                                                                                                                                    >  1:55294/1‑301 (MQ=255)`  
                                                                                 `gTTTGCAGGAAGGGATTGCCCGCTCATGGATGAACAGCTACGTCTCCAGACCTGAGGTCAAGGCCAGAGTCGATGAGATGCTTAAGGAATTACACGGGGTGAAGGAAGTAACACCAGAGATGGTAGAGAAGTACGCTATGGATAAGGCTTATGGTATCTCCCACTCAGACCAGTTCACCAACAGTTCCATAATAGAAGAGAACATTGAGGGCTTAGTAGTTATCGAGAATAACTCATTCCTTGAGGCACGTCACTTGTTTGATTCGGACCTGTctcttatacacatctccgagcccacgag                                                                                                                                                                                                                             >  1:21845/1‑274 (MQ=255)`  
                                                                                        `ggAAGGGATTGCCCGCTCATGGATGAACAGCTACGTCTCCAGACCTGAGGTCAAGTACAGAGTCGATGAGATGCTTAAGGAATTACACGGGGTGAAGGAAGTAACACCAGAGATGGTAGAGAAGTACGCTATGGATAAGGCTTATGGTATCTCCCACCCAGACCAGTTCACCAACAGTTCCATAATAGAAGAGAACATTGAGGGCTTAGTAGGTATCGCTAATAACTCATTCCTTGAAGCACGTAACTTGTTTGATTCGGACCTATCCATCAATATGCCAGCCGGACAGCACTTCACAGTg                                                                                                                                                                                                                      >  1:402455/1‑301 (MQ=255)`  
                                                                                            `gggATTTCCCGCTCATGGATGAACAGCTACGTCTCCAGACCTGAGGTCAAGGCCAGAGTCTATGAGATGCTTAAGGAATTACACGGGGTGAAGGAAGTAACACCAGAGATGGTAGAGAAGTACGCTATGGATAAGGCTTATGGTATCTCCCACTCAGACCAGTTCACCAACAGTTCCATAATAGAAGAGAACATTGAGGGCTTAGTAGTTATCGAGAATAACTCATTCCTTGAGGCACGTAACTTGTTTGATTCGGACCTATCCATCACTATGCCAGACGGACAGCAATTCTCAGTGAATg                                                                                                                                                                                                                  >  1:345068/1‑301 (MQ=255)`  
                                                                                              `gATTGCCCGCTCATGGAGGAACAGTTAAGTCTCCAGACCGGAGGGCAAGGCAAGAGTCGATGAGATGCTTAAGGAATTACACGGGGTGAAGGAAGTAACACCAGAGATGGTAGAGAAGTACGCTATGGATAAGGCTTATGGTATCTCCCACTCAGACCAGTTCACCAACAGTTCCATAATAGAAGAGAACATTGAGGGCTTAGTAGTTATCGAGAATAACTCATTCCTTGAGGCACGTAACTTGTTTGATTCGGACCTATCCATCACTATGCCAGACGGACAGCAATTCTCAGTGAATGAc                                                                                                                                                                                                                <  1:803579/301‑1 (MQ=255)`  
                                                                                               `aTTGCCCGCTCATGGATGAACAGCTACGTCTCCAGACCTGAGGTCAAGGCCAGAGTCGATGAGATGCTTAAGGAATTACACGGGGTGAAGGAAGTAACACCAGAGATGGTAGAGAAGTACGCTATGGATAAGGCTTATGGTATCTCCCACTCAGACCAGTTCACCAACAGTTCCATAATAGAATAGAACATTGAGGGCTTAGTAGGTATCGAGAATAACTCATTCCTTGAGGCACGTAACTTGTTTGATTCTGACCTATCCATCACTATGCCAGACGGACAGCAATTCTCAGTGAATGAcc                                                                                                                                                                                                               <  1:179043/301‑1 (MQ=255)`  
                                                                                                      `gCTCATGGATGAACAGCTACGTCTCCAGACCTGAGGTCAAGGCCAGAGTCGATGAGATGCTTAAGGAATTACACTGGGTGAAGGAAGTAACACCAGAGATGGTAGAGAAGTACGCTATGGATAAGGCTTATGGTATCTCCCACTCAGACCAGTTCACCAACAGTTCCATAATAGAAGAGAACATTGAGGGCTTAGTAGGTATCGAGAATAACTCATTCCTTGAGGCACGTAACTTGTTTGATTCGGACCTATCCATCACTATGCCAGACGGACAGCAATTCTCAGTGAATGACCTAAGGGa                                                                                                                                                                                                        >  1:413078/1‑301 (MQ=255)`  
                                                                                                       `cTCATGGATGAACAGCTACGTCTCCAGACCTGAGGTCAAGGCCAGAGTCGATGAGATGCTTAAGGAATTACACGGGGTGAAGGAAGTAACACCAGAGATGGTAGAGAAGTACGCTATGGATAAGGCTTATGGTATCTCCAACTCAGACCAGTTCACCAACAGTTCCATAATAGAAGAGAACATTGAGGGCTTAGTAGGTATCGAGAATAACTCATTCCTTGAGGCACGTAACTTGTTTGATTCGGACCTATCCATCACTATGCCAGACGGACAGCAATTATCAGTGAATGACCTAAGGGAc                                                                                                                                                                                                       <  1:547951/301‑1 (MQ=255)`  
                                                                                                                `atgtgtgtaagagacaggACCTGAGGTCAAGGCCAGAGTCGATGAGATGCTTAAGGAATTACACGGGGTGAAGGAAGTAACACCAGAGATGGTAGAGAAGTACGCTATGGATAAGGCTTATGGTATCTCACACTCAGACCAGTTCACCAACAGTTCCATAATAGAAGAGAACATTGAGGGCTTAGTAGGTATCGAGAATAACTCATTCCTTGAGGCACGTAACTTGTTTGATTCGGACCTATCCATCACTATGCCAGACGGACAGCAATTCTCAGTGAATGACCTAAGGGACTTCAATATg                                                                                                                                                                                              <  1:476380/284‑1 (MQ=255)`  
                                                                                                                 `gatgtgtataagagacagccTGAGGTCAATGCCAGAGTCGATGAGATGCTTAAGGAATTACACGGGGTGAAGGAAGTAACACCAGAGATGGTAGAGAAGTACGCTATAGATAAGGCTTATGGTATCTCCCACTCAGACCAGTTCACCAACAGTTCCATAATAGAAGAGAACATTGAGGGCTTAGTAGGTATCGAGAATAACTAATTCATTGAGGCACGTAACTTGTTTGATTCGGACCTATCCATCACTATGCCAGACGGACAGCAATTCTCAGTGAATGACCTAAGGGACTTCGATATGt                                                                                                                                                                                             <  1:268967/283‑1 (MQ=255)`  
                                                                                                                   `cAGCTACGTCTCCAGACCTGAGGTCAAGGCCAGAGTCGATGAGATGCTTAAGGAATTACACGGGGTGAAGGAAGTAACACCAGAGATGGTAGAGAAGTACGCTATGGATAAGGCTTATGGTATCTCCCACTCAGACCAGTTCACCAACAGTTCCATAATAGAAGAGAACATTGAGGGCTTAGTAGGTATCGAGAATAACTCATTCCTTGAGGCACGTAACTTGTTTGATTCGGACCTATCCATCACTATGCCAGACGGACAGCAATTCTCAGTGAATGACCTAAGGGACTTCGATATGTTc                                                                                                                                                                                           >  1:754405/1‑301 (MQ=255)`  
                                                                                                                   `cAGCTACGTCGCCAGACCGGAGGTCAAGGCCAGAGTCGATGAGATGCTTAAGGAATTACACGGGGTGAAGGAAGTAAAACCAGAGATGGTAGAGAAGTACGCTATGGAGAAGGCTTATGGTAGCTGCCACGCAGACCAGTTCACCAACAGTTCCATAATAGAAGAGAACATTGAGGGCTTAGTAGGTATCGAGAATAACTCAGTCCTTGAGGCACGTAACTTGTTTGATTCGGACCTAGCCATCACTATGCCAGACGGACAGCAATTCTCAGTGAATGACCTAAGGGACTTCGATATGTTc                                                                                                                                                                                           <  1:404248/301‑1 (MQ=255)`  
                                                                                                                         `cGTCTCCAGACCTGAGGTCAAGGCCAGAGTCGATGAGATGCTTAAGGAATTACACGGGGTGAAGGAAGTAACACCAGAGATGGTAGAGAAGTACGCTATGGATAAGGCTTATGGTATCTCACACTCAGACCAGTTCACCAACAGTTCCATAATAGAAGAGAACATTGAGGGCTTAGTAGTTATCGAGAATAACTCATTCCTTGAGGCACGTAACTTGTTTGATTCGGACCTATCCATCACTATGCCAGACGGACAGCAATTCTCAGTGAATGACCTAAGGGACTTCGATATGTTCCGcatc                                                                                                                                                                                     >  1:11569/1‑301 (MQ=255)`  
                                                                                                                            `ctcCAGACCTGAGGTCAATGCCAGAGTCGATGAGATGCTTAAGGAATTACACGGGGTGAAGGAAGTAACACCAGAGATGGTAGAGAAGTACGCTATGGATAAGGCTTATGGTATCTCCCACTCAGACCAGTTCACCAACAGTTCCATAATAGAAGAGAACATTGAGGGCTTAGTAGGTATCGAGAATAACTCATTCCTTGAGGCACGTAACTTGTTTGATTCGGACCTATCCATCACTATGCCAGACGGACAGCAATTCTCAGTGAATGACCTAAGGGACTTCGATATGTTCCGcatcatt                                                                                                                                                                                  >  1:9363/1‑300 (MQ=255)`  
                                                                                                                            `ctcCAGACCTGAGGTCAAGGCCAGAGTCGATGAGATGCTTAAGGAATTACACGGGGTGAAGGAAGTAACACCAGAGATGGTAGAGAAGTACGCTATTGATAAGGCTTATGGTATCTCCCACTCAGACCAGTTCACCAACAGTTCCATAATAGAAGAGAACATTGAGGGCTTAGTAGGTATCGAGAATAACTCATTCCTTGAGGCACGTAACTTGTTTGATTCGGACCTATCCATCACTATGCCAGACGGACAGCAATTCTCAGTGAATGACCTAAGGGACTTCGATATGTTCCGCATCATg                                                                                                                                                                                  >  1:382402/1‑301 (MQ=255)`  
                                                                                                                             `tcCAGACCTGAGGTCAAGGCCAGAGTCGATGAGATGCTTAAGGAATTACACGGGGTGAAGGAAGTAACACCAGAGATGGTAGAGAAGTACGCTATGGATAAGGCTTATGGTATCTCCCACTCAGACCAGTTCACCAACAGTTCCATAATAGAAGAGAACATTGAGAGCTTAGTAGGTATCGAGAATAACTCATTCCTTGAGGCACGTAACTTGTTTGATTCGGACCTATCCATCACTATGCCAGACGGACAGCAATTCTCAGTGAATGACCTAAGGGACTTCGATATGTTCCGCATCATGc                                                                                                                                                                                 <  1:121254/301‑1 (MQ=255)`  
                                                                                                                                `aGACCTGAGGTCAAGGCCAGAGTCGATGAGATGCTTAAGGAATTACACGGGGTGAAGGAAGTAACACCAGAGATGGTAGAGAAGTACGCTATGGATAAGGCTTATGGTATCTTCCACTCAGACCAGTTCACCAACAGTTCCATAATAGAAGAGAACATTGAGGGCTTAGTAGGTATCGAGAATAACTCATTCCTTGAGGCACGTAACTTGTTTGATTCGGACCTATCCATCACTATGCCAGACGGACAGCAATTCTCAGTGAATGACCTAAGGGACTTCGATATGTTCCGCATCATGCCAg                                                                                                                                                                              <  1:388490/301‑1 (MQ=255)`  
                                                                                                                                           `cAAGGCCAGAGTCGATGAGATGCTTAAGGAATTACACGGGGTGAAGGAAGTAACACCAGAGATGGTAGAGAAGTACGCTATGGATAAGGCTTATGGTATCTCCCACTCAGACCAGTTCACCAACAGTTCCATAATAGAAGAGAACATTGAGGGCTTAGTAGGTATCGAGAATAACTCATTCCTTGAGGCACGTAACTTGTTTGATTCGGACCTATCCATCACTATGCCAGACGGACAGCAATTCTCAGTGAATGACCTAAGGGACTTCGATATGTTCCGCATCATGCCAGCGTATGAccgc                                                                                                                                                                   <  1:17399/301‑1 (MQ=255)`  
                                                                                                                                              `ggCCAGAGTCGATGAGATGCTTAAGGAATTACACGGGGTGAAGGAAGTAACACCAGAGATGGTAGAGAAGTACGCTATGGATAAGGCTTATGGTATCTCCCACTCAGACCAGTTCACCAACAGTTCCATAATAGAAGAGAACATTGAGGGCTTAGTAGGTATCGAGAATAACTCATTCCTTGAGGCACGTAACTTGTTTGATTCGGACCTATCCATCACTATGCCAGACGGACAGCAATTCTCAGTGAATGACCTAAGGGACTTCGATATGTTCCGCATCATGCCAGCGTATGACCGCCgt                                                                                                                                                                >  1:687629/1‑301 (MQ=255)`  
                                                                                                                                                        `gATGAGATGCTTAAGGAATTACACGTGGTGAAGGAAGTAACACCAGAGATGGTAGAGAAGTACGCTATGGATAAGGCTTATGGTATCTCCCACTCAGACCAGTTCACCAACAGTTCCATAATAGAAGAGAACATTGATGGCTTAGTAGGTATCGAGAATAACTCATTCCTTGAGGCACGTAACTTGTTTGATTCCGACCTATCCATCACTATGCCAGACGGACAGCAATTCTCAGTGAATGACCTAAGGGACTTCGATATGTTCCGCATCATGCCAGCGTATGACCGCCGTGTCAATCGTg                                                                                                                                                      >  1:58461/1‑301 (MQ=255)`  
                                                                                                                                                               `cggagatgtgtataagagacaggaaggaagTAACACCAGAGATGGTAGAGAAGTACGCTATGGATAAGGCTTATGGTATCTCCCACTCAGACCAGTTCACCAACAGTTCCATAATAGAAGAGAACATTGAGTGCTTAGTAGGTATCGAGAATAACTCATTCCTTGAGGCACGTAACTTGTTTGATTCGGACCTATCCATCACTATGCCAGACGGACAGCAATTCTCAGTGAATGACCTAAGGGACTTCGATATGTTCCGCATCATGCCAGCGTATGACCGCCGTGTCAATGGTGGCATCGc                                                                                                                                               <  1:816099/279‑1 (MQ=255)`  
                                                                                                                                                                         `cgtgggctcggagatgtgtataagagacaggaTGGTAGAGAAGTACGCTATGGATAAGGCTTATGGTATCTCCCACTCAGACCAGTTCACCAACAGTTCCATAATAGAAGAGAACATTGAGGGCTTAGTAGGTATCGAGAATAACTCATTCCTTGAGGCACGTAACTTGTTTGATTCGGACCTATCCATCACTATGCCAGACGGACAGCAATTCTCAGTGAATGACCTAAGGGACTTCGATATGTTCCGCATCATGCCAGCGTATGACCGCCGTGTCAATGGTGACATCGCCATCATgggg                                                                                                                                     <  1:449527/271‑1 (MQ=255)`  
                                                                                                                                                                         `agagacaggggTGAAGGAAGTAACACCAGAGATGGTAGAGAAGTACGCTATGGATAAGGCTTATGGTATATCCCACTCAGACCAGTTCACCAACAGTTCCATAATAGAAGAGAACATTGAGGGCTTAGTAGGTATCGAGAATAACTCATTCCTTGAGGCACGTAACTTGTTTGATTCGGACCTATCCATCACTATGCCAGACGGACAGCAATTCTCAGTGAATGACCTAAGGGACTTCGATATGTTCCGCATCATGCCAGCGTATGACCGCAGTGTCAATGGTGACATCGCCATCATgggg                                                                                                                                     <  1:223960/294‑1 (MQ=255)`  
                                                                                                                                                                         `aTTACACTGGGTGAAGGAAGTAACACCAGAGATGGTAGAGAAGTACGCTATGGATAAGGCTTATGGTATCTCCCACTCAGACCAGTTCACCAACAGTTCCATAATAGAAGAGAACATTGAGGGCTTAGTAGTTATCGAGAATAACTCATTCCTTGAGGCACGTAACTTGTTTGATTCGGACCTATCCATCACTATGCCAGACGGACAGCAATTCTCAGTGAATGACCTAAGGGACTTCGATATGTTCCGCATCATGCCAGCGTATGACCGCCGTGTCAATGGTGACATCGCAATCATgggg                                                                                                                                     <  1:821330/301‑1 (MQ=255)`  
                                                                                                                                                                         `aTTACACGGGGTGAAGGAAGTAACACCAGAGATGGTAGAGAAGTACACTATGGATAAGGCTTATGGTATCTCCCACTCAGACCAGTTCACCAACAGTTCCATAATAGAAGAGAACATTGAGGGCTTAGTAGGTATCGAGAATAACTCATTCCTTGAGGCACGTAACTTGTTTGATTCGAACCTAACCATCACTATGCCAGACGGACAGCAATTCTCAGTGAATGACCTAAGGGACTTCGATATGTTCCGCATCATGCCAGCGTATGACAGCCGTGTCAATCGTGACATCACCATCATgggg                                                                                                                                     <  1:111313/301‑1 (MQ=255)`  
                                                                                                                                                                                 `gggTGAAGGAAGTAACACCAGAGATGGTAGAGAAGTACGCTATGGATAAGGCTTATGGTATCTCCCACTCAGCCCAGTTCACCAACAGTTACATAATAGAAGAGAACATTGAGGGCTTAGTAGTTATCGAGAACAACGCATTCCTTGAGGCACGTAACTTGTTTGATTCGGACCTATCCATCACTATGCCAGACGGACAGCAATTCTCAGTGAATGACCTAAGGGACTTCGATATGTTCCGCATCATGCCAGCGTATGACCGCCGTGTCAATGGTGACATCGCAATCATGGGGTCTACTgg                                                                                                                             <  1:773906/301‑1 (MQ=255)`  
                                                                                                                                                                                 `gggTGAAGGAAGTAACACCAGAGATGGTAGAGAAGTACGCTATGGATAAGGCTTATGGTATCTCCCACTCAGACCAGTTCACCAACAGTTCCATAATAGAATAGAACATTGAGGGCTTAGTAGGTATCGAGACTAACTCATTCCTTGAGGCACGTAACTTGTTTGATTCGGACTTATCCATCACTATGCCAGACGGCCAGCAATTCTCAGTGAATGACCTAAGGGACTTCGATATGTTCCGCATCATGCCAGCGTATGATCTCAGTGTCACTCGCTCCATCGCCATCATGGGGTCTACTgt                                                                                                                             >  1:106057/1‑300 (MQ=255)`  
                                                                                                                                                                                  `ggTGAAGGAAGTAACACCAGAGATGGTAGAGAAGTACGCTATGGATAAGGCTTATGGTATCTCCCACTCAGACCAGTTCACCAACAGTTCCATAATAGAAGAGAACATTGAGGGCTTAGTAGGTATCGAGAATAACTCATTCCTTGAGGCACGTAACTTGTTTGATTCGGACCTATCCATCACTATGCCAGACGGACAGCAATTCTAAGTGAATGACCTAAGGGACTTCGATATGTTCCACATCATGCCAGCGTATGACCGCCGTGTCAATGGTGACATCGCAATCATGGGGTATACTGGt                                                                                                                            >  1:214913/1‑301 (MQ=255)`  
                                                                                                                                                                                        `ggaagTAACACCAGAGATGGTAGAGAAGTACGCTATGGATAAGGCTTATGGTATCTCCCACTCAGACCAGTTCACCAACAGTTCCATAATAGAAGAGAACATTGAGGGCTTAGTAGGTATCGAGAATAACTCATTCCTTGAGGCACGTAACTTGTTTGATTCGGACCTATCCATCACTATGCCAGACGGACAGCAATTCTCATTGAATGACCTAAGGGACTTCGATATGTTCCGCATCATGCCAGTGTATGACCGCTGTGTCAATGGTGACATCGCCATCATggggctgtctcttatacac                                                                                                                      >  1:133924/1‑286 (MQ=255)`  
                                                                                                                                                                                          `ctcggagatgtgtataagagacagaGTACGCTATGGATAAGGCTTATGGTATCTCCCACTCAGACCAGTTCACCAACAGTTCCATAATAGAAGAGAACATTGAGGGCTTAGTAGGTATCGAGAATAACTCATTCCTTGAGGCACGTAACTTGTTTGATTCGGACCTATCCATCACTATGCCAGACGGACAGCAATTCTCAGTGAATGACCTAAGGGACTTCGATATGTTCCGCATCATGCCAGCGTATGACCGCCGTGTCAATGGTGACATCGCCATCATGGGGTCTACTGGTAAAACCAc                                                                                                                    <  1:456422/277‑1 (MQ=255)`  
                                                                                                                                                                                          `aagTAACACCAGAGATGGTAGAGAAGTACGCTATGGATAAGGCTTATGGTATCTCCCACTCAGACCAGTTCACCAACAGTTCCATAATAGAAGAGAACATTGAGGGCTTAGTAGGTATCGAGAATAACTCATTCCTTGAGGCACGTAACTTGTTTGATTCGGACCTATCCATCACTATGCCAGACGGACAGCAATTCTCAGTGAATGACCTAAGGGACTTCGATATGTTCCGCATCATGCCAGCGTATGACCGCAGTGTCAATCGTGACATCGCCATCATGGGGTCTACTGGTAAAACCAc                                                                                                                    >  1:291885/1‑301 (MQ=255)`  
                                                                                                                                                                                           `agTAACACCAGAGATGGTAGAGAAGTACGCTATGGATAAGGCTTATGGTATCTCCCACTCAGACCAGTTCACCAACAGTTCCATAATAGAAGATAACATTTAGGGCTTAGTAGGTATCGAGAATAACTCATTCCTTGAGGCACGTAACATGTTTGATTCGGACCTATCCATCACTATGCCAGACGGACAGCAATTCTCAGTGAATGACCTAAGGGACTTCAATATGTTCCGCATCATGCCAGCGTATGACCGCCGTGTCAATCGTGACATCGCCATCATggggctgtctcttatacacatc                                                                                                                   >  1:697885/1‑283 (MQ=255)`  
                                                                                                                                                                                                    `agagATGGTAGAGAAGTACGCTATGGATAAGGCTTATGGTATCTCCCACTCAGACCAGTTCACCAACAGTTCCATAATAGAAGAGAACATTGAGGGCTTAGTAGTTATCGAGAATAACTCATTCCTTGAGGCACGTAACTTGTTTGATTCTGACCTATCCATCACTATGCCAGACGGACAGCAATTCTCAGTGAATGACCTAAGGGACTTCGATATGTTCCGCATCATGCCAGCGTATGACCGCCGTGTCAATGGTGACATCGCCATCATGGGGTCTACTGGTAAAACCACTAAGGAACtt                                                                                                          <  1:638661/301‑1 (MQ=255)`  
                                                                                                                                                                                                      `agaTGGTAGAGAAGTACGCTATGGATAAGGCTTATGGTATCTCCCACTCAGACCAGTTCACCAACAGTTCCATAATAGAAGAGAACATTGATGGCTTAGTAGGTATCGAGAATAACTCATTCCTTGAGGCACGTAACTTGTTTGATTCGGACCTATCCATCACTATGCCAGACGGACAGCAATTCTCAGTGAATGACCTAAGGGACTTCGATATGTTCCGCATCATGCCAGCGTATGACCGCCGTGTCAATCGTGACATCGCCATCATGGGGTCTACTGGTAAAACCACTAAGGAACTTaa                                                                                                        <  1:479158/301‑1 (MQ=255)`  
                                                                                                                                                                                                         `taagagacaggTACGCTATGGATAAGGCTTATGGTATCTCCCACTCAGACCAGTTCACCAACAGTTCCATAATAGAAGAGAACATTGAGGGCTTAGTAGGTATCGAGAATAACTCATTCCTTGAGGCACGTAACTTGTTTGATTCGGACCTATCCATCACTATGCCAGACGGACAGCAATTCTCAGTGAATGACCTAAGGGACTTCGATATGTTCCGCATCATGCCAGCGTATGACCGCCGTGTCAATCGTGACATCGCCATCATGGGGTCTACTGGTAAAACCACTAAGGAACTTAAGGa                                                                                                     <  1:278640/291‑1 (MQ=255)`  
                                                                                                                                                                                                         `tGGTAGAGAAGTACGCTATGGATAAGGCTTATGGTATCTCCCACTCAGACCAGTTCACCAACAGTTCCATAATAGAAGAGAACATTGAGGGCTTAGTAGGTATCGAGAATAACTCATTCCTTGAGGCACGTAACTTGTTTGATTCGGACCTATCCATCACTATGCCAGACGGACAGCAATTCTCAGTGAATGACCTAAGGGACTTCGATATGTTCCGCATCATGCCAGCGTATGACCGCCGTGTCAATCGTGACATCGCCATCATGGGGTCTACTGGTAAAACCACTAAGGAACTTAAGGa                                                                                                     <  1:336840/301‑1 (MQ=255)`  
                                                                                                                                                                                                         `gctcggagatgtgtataagagacagggCTTATGGTATCTCCCACTCAGACCAGTTCACCAACAGTTCCATAATAGAAGAGAACATTGAGGGCTTAGTAGGTATCGAGAATAACTCATTCCTTGAGGCACGTAACTTGTTTGATTCGGACCTATCCATCACTATGCCAGACGGACAGCAATTCTCAGTGAATGACCTAAGGGACTTCGATATGTTCCGCATCATGCCAGCGTATGACCGCCGTGTAAATCGTGACATCGCCATCATGGGGTCTACTGGTAAAACAACTAAGGAACTTGAGGa                                                                                                     <  1:765597/276‑1 (MQ=255)`  
                                                                                                                                                                                                           `gTAGAGAAGTACGCTATGGATAAGGCTTATGGTATCTCCCACTCAGACCAGTTCACCAACAGTTCCATAATAGAAGAGAACATTGATGGCTTAGTAGGTATCGAGAATAACTCATTCCTTGAGGCACGTAACTTGTTTGATTCGGACCTATCCATCACTATGCCAGACGGACAGCAATTCTCAGTGAATGACCTAAGGGACTTCGATATGTTCCGCATCATGCCAGCGTATGACCGCAGTGTCAATGGTGACATCGCCATCATGGGGTCTACTGGTAAAACCACTAAGGAACTTAAGGact                                                                                                   >  1:241941/1‑299 (MQ=255)`  
                                                                                                                                                                                                                `gaAGTACGCTATGGATAAGGCTTATGGTATCTCCCACTCAGACCAGTTCACCAACAGTTCCATAATAGAAGAGAACATTGAGGGCTTAGTAGGTATCGAGAATAACTCATTCCTTGAGGCACGTAACTTGTTTGATTCGGACCTATCCATCACTATGCCAGACGGACAGCAATTCTCAGTGAATGACCTAAGGGACTTCGATATGTTCCGCATCATGCCAGCGTATGACCGCCGTGTCAATCGTGACATCGCCATCATGGGGTCTACTGGTAAAACCACTAAGGAACTTAAGGATGAGAtt                                                                                              >  1:631043/1‑301 (MQ=255)`  
                                                                                                                                                                                                                  `aGTACGCTATGGATAAGGCTTATGGTATCTCCCACTCAGACCAGTTCACCAACAGTTCCATAATAGAAGAGAACATTGAGGGCTTAGTAGTTATCGAGAATAACTCATTCCTTGAGGCACGTAACTTGTTTGATTCGAACCTATCCATCACTATGCCAGACGGACAGCAATTCTCAGTGAATGACCTAAGGGACTTCGATATGTTCCGCATCATGCCAGCGTATGACCGCCGTGTCAATGGTGACATCGCCATCATGGGGTCTACTGGTAAAACCACTAAGGAACTTAAGGATGAGAtttt                                                                                            >  1:331951/1‑301 (MQ=255)`  
                                                                                                                                                                                                                   `gTACGCTATGGATAAGGCTTATGGTATCTCCCACTCAGACCAGTTCACCAACAGTTCCATAATAGAAGAGAACATTGAGGGCTTAGTAGGTATCGAGAATAACTCATTCCTTGAGGCACGTAACTTGTTTGATTCGGACCTATCCATCACTATGCCAGACGGACAGCAATTCTCAGTGAATGACCTAAGGGACTTCGATATGTTCCGCATCATGCCAGCGTATGACCGCCGTGTCAATCGTGACATCGCCATCATGGGGTCTACTGGTAAAACCACTAAGGAACttctgtctcttatacac                                                                                           >  1:614375/1‑286 (MQ=255)`  
                                                                                                                                                                                                                      `cGCTATGGATAAGGCTTATGGTATCTCCCACTCAGACCAGTTCACCAACAGTTCCATAATAGAAGAGAACATTGAGGGCTTAGTAGGTATCGAGAATAACGCATTCCTTGAGGCACGTAACTTGTTTGATTCGGACCTATCCATCACTATGCCAGACGGACAGCAATTCTCAGTGAATGACCTAAGGGACTTCGATATGTTCTGCATCATGCCAGCGTATGACCGCCGTGTCAATGGTGACATCGCCATCATGGGGTCTACTGGTAAAACCACTAAGGAACTTAAGGATGAGATTTTGGct                                                                                        <  1:572018/301‑1 (MQ=255)`  
                                                                                                                                                                                                                          `aTGGATAAGGCTTATGGTATCTCCCACTCAGACCAGTTCACCAACAGTTCCATAATAGAAGAGAACATTGAGGGCTTAGTAGTTATCGAGAATAACTCATTCCTTGAGGCACGTAACTTGTTTGATTCGGACCTATCCATCACTATGCCAGACGGACAGCAATTCTCAGTGAATGACCTAAGGGACTTCGATATGTTCCGCATCATGCCAGCGTATGACCGCCGTGTCAATGGTGACATCGCCATCATGGGGTCTACTGGTAAAACCACTAAGGAACTTAAGGATGAGATTTTGGCTCTCa                                                                                    >  1:310058/1‑301 (MQ=255)`  
                                                                                                                                                                                                                          `aTGGATAAGGCTTATGGTATCTCCCACTCAGACCAGTTCACCAACAGTTCCATAATAGAAGAGAACATTGAGGGCTTAGTAGGTATCGAGAATAACTCATTCCTTGAGGCACGTAACTTGTTTGATTCGGACCTATCCATCACTATGCCAGACGGACAGCAATTCTCAGTGAATGACCTAAGGGACTTCGATATGTTCCGCATCATTCCAGCGTATGACCGCCGTGTCAATAGTTACATCGCCATCATGGGGTCTACTTGTAAAACCATTAGGAAATTTagtgtgagggttttgccttcca                                                                                    >  1:782942/1‑280 (MQ=255)`  
                                                                                                                                                                                                                                 `aGGCTTATGGTATCTACCACTCAGACCAGTTCACCAACAGTTCCATAATAGAAGAGAACATTGAGGGCTTAGTAGGTATCGAGAATAACTCATTCCTTGAGGCACGTAACTTGTTTGATTCGGACCTATCGATCACTATGCCAGACAGACAGCAATTCTCAGTGAATGACCTAAGGGACTTCGATATGTTCCGCATCATGCCAGCGTATGAACGCCGTGTCACTCGTTAACTCGACATACACGGGTCTACTGGTCAAACCACTCAGGAACTTAAGGATGCAATTTTGGCTCTCACAGCGaa                                                                             >  1:649781/1‑301 (MQ=255)`  
                                                                                                                                                                                                                                  `ggCTTATGGTATCTCCCACTCAGACCAGTTCACCAACAGTTCCATAATAGAAGAGAACATTGAGGGCTTAGTAGGTATCGAGAATAACTCATTCCTTGAGGCACGTAACTTGTTTGATTCGGACCTATCCATCACTATGCCAGACGGACAGCAATTCTCAGTGAATGACCTAAGGGACTTCGATATGTTCCGCATCATGCCAGCGTATGACCGCCGTGTCAATCGTGACATCGCCATCATGGGGTCTACTGGTAAAACCACTAAGGAACTTAAGGactgtctcttatacacatctccgagc                                                                            >  1:285775/1‑276 (MQ=255)`  
                                                                                                                                                                                                                                          `gTATCTCCCACTCAGACCAGTTCACCAACAGTTCCATAATAGAAGAGAACATTGAGGGCTTAGTAGGTATCGAGAATAACTCATTCCTTGAGGCCCGTAACTTGTTTGATTCGGACCTATCAATCACTATGCCAGACGGACAGCAATTCTCAGTGAATGACCTACGGGACTTCGATATGTTCCGCATCATGCCAGCGTATGACCGCCGTGTCAATAGTGACATCGCCATCATGGGGTCTACTGGTAAAACCACTAAGGAACTTAAGGATGAGATTTTGGCTCTCACAGCGAAAGCTGAggg                                                                    >  1:235571/1‑301 (MQ=255)`  
                                                                                                                                                                                                                                              `ctcCCACTCAGACCAGTTCACCAACAGTTCCATAATAGAAGAGAACATTGAGGGCTTAGTAGTTATCGAGAATAACTCATTCCTTGAGGCACGTAACTTGTTTGATTCGGACCTATCCATCACTATGCCAGACGGACAGCAATTCTCAGTGAATGACCTAAGGGACTTCGATATGTTCCGCATCATGCCAGCGTATGACCGCCGTGTCAATGGTGACATCGCCATCATGTGGTCTACTGGTAAAACCACTAAGGAACTTAAGGATGAGATTTTGGCTCTCAAAGCGAAAGCTGAGGGAGAc                                                                >  1:747410/1‑301 (MQ=255)`  
                                                                                                                                                                                                                                                   `aCTCAGACCAGTTCACCAACAGTTCCATAATAGAAGAGAACATTGAGGGCTTAGTAGGTATCGAGAATAACTCTTTCCTTGAGGCACCTATCTTGTTTGTTTCGGCCCTATCCTTCCCTTTGCCAGACTGACTCCAATTCTCAGTTCTTGACCTACTTGCCTTCGATATGTTCCGCATCATGCCATCTTCTGACCGCCGTGTCAATGGTGACATCGCCATCTTGGGTTCTACTGGTAAAACCACTAAGGAATTTAAGGATGATATTTTGGCTCTCATAGCGAAAGCTGggggcgcctgttt                                                           >  1:317993/1‑292 (MQ=255)`  
                                                                                                                                                                                                                                                   `aCTCAGACCAGTTCACCAACAGTTCCATAATAGAAGAGAACATTGAGGGCTTAGTAGGTATCGAGAATAACTCATTCCTTGAGGCACGTAACTTGTTTGATTCGGACCTATCCATCACTATGCCAGACGGACAGCAATTCTCAGTGAATGACCTAAGGGACTTCGATATGTTCCGCATCATGCCAGCGTATGACCGCCGTGTCAATCGTGACATCGCCATCATGGGGTCTACTGGTAAAACTACTAAGGAACTTAAGGATGAGATTTTGGCTCTCAAAGCGAAAGCTGAGGGAGACGGTaa                                                           >  1:604065/1‑301 (MQ=255)`  
                                                                                                                                                                                                                                                       `aGACCAGTTCACCAACAGTTCCATAATAGAAGAGAACATTGAGGGCTTAGTAGGTATCGAGAATAACTCATTCCTTGAGGCACGTAACTTGTTTGATTCGGACCTATCCATCACTATGCCAGACGGACAGCAATTCTCAGTGAATGACCTAAGGGACTTCGATATGTTCCGCATCATGCCAGCGTATGACCGCCGTGTCAATGGTGACATCGCCATCATGGGGTCTACTGGTAAAACCACTAAGGAACTTGAGGATGAGATTTTGGCTCTCAAAGCGAAAGCTGAGGGAGACGGTaagaag                                                       <  1:101962/301‑1 (MQ=255)`  
                                                                                                                                                                                                                                                       `aGACCAGTTCACCAACAGTTCCATAATAGAAGAGAACATTGAGGG‑TTAGTAGGTATCGAGAATAACTCATTCCTTGAGGCACGTAACTTGTTTGATTCGGACCTATCCATCACTATGCCAGACGGACAGCAATTCTCAGTGAATGACCTAAGGGACTTCGATATGTTCCGCATCATGCCAGCGTATGACCGCCGTGTCAATCGTGACATCGCCATCATGGGGTATACTGGTAAAACCACTAAGAAACTTAAGGATGAGATTTTGGCTCTCAAAGCGAAAGCTGAGGGAGACGGTAagaaga                                                      <  1:197413/301‑1 (MQ=255)`  
                                                                                                                                                                                                                                                         `acaggTTCACCAACAGTTCCATAATAGAAGAGAACATTGAGGGCTTAGTAGGTATCGAGAATAACTCATTCCTTGAGGCACGTAACTTGTTTGATTCGGACCTATCCATCACTATGCCAGACGGACGGCAATTCTCAGTGAATGACCTAAGGGACTTCGATATGTTCCGCATCATGCCAGCGTATGACCGCCGTGTCAATCGTGACATCGCCATCATGGGGTCTACTGGTAAAACCACTAAGGAACTTAAGGATGAGATTTTGGCTCTCAAAGCGAAAGCTGAGGGAGACGGTAAGAAGAc                                                     <  1:501109/297‑1 (MQ=255)`  
                                                                                                                                                                                                                                                             `gTTCACCAACAGTTCCATAATAGAAGAGAACATTGAGGGCTTAGTAGTTATCGAGAATAACTCATTCCTTGAGGCACGTAACTTGTTTGATTCGGACCTATCCATCACTATGCCAGACGGACAGCAATTCTCAGTGAATGACCTAAGGGACTTCGATATGTTCCGCATCATGCCAGCGTATGACAGCCGTGTCAATGGTGACATCGCCATCATGGGGTCTACTGGTAAAACCACTAAGGAACTTAAGGATGAGATTTTGGCTCTCAAAGCGAAAGCTGAGGGAGACGGTAAGAAGACTGGc                                                 >  1:433243/1‑301 (MQ=255)`  
                                                                                                                                                                                                                                                             `gTTCACCAACAGTTCCATAATAGAAGAGAACATTGAGGGCTTAGTAGGTATCGAGAATAACTCATTCCTTGAGGCACGTAACTTGTTTGATTCGGACCTATCCATCACTATGCCAGACGGACAGCAATTCTCAGTGAATGACCTAAGGGACTTCGATATGTTCCGCATCATGCCAGCGTATGACCGCCGTGTCAATGGTGACATCGCCATCATGGGGTCTACTGGTAAAACCACTAAGGAACTTGAGGATGAGATTTTGGCTCTCAAAGCGAAAGCTGAGGGAGACGGTAAGAAGACTggg                                                 >  1:574659/1‑300 (MQ=255)`  
                                                                                                                                                                                                                                                                `ctcggagatgtgtataagaGACGGGAACATTGAGGGCTTAGTAGGTATCGAGAATAACTCATTCCTTGAGGCACGTAACTTGTTTGATTCGGACCTATCCATCACTATGCCAGACGGACAGCAATTCTCAGTGAATGACCTAAGGGACTTCGATATGTTCCGCATCATGCCAGCGTATGACCGCCGTGTCAATCGTGACATCGCCATCATGGGGTCTACTGGTAAAACCACTAAGGAACTTAAGGATGAGATTTTGGATCTCAAAGCGAAAGCTGAGGGAGACGGTAAGAAGACTGGCGAg                                              <  1:158836/288‑1 (MQ=255)`  
                                                                                                                                                                                                                                                                `cACCAACAGTTCCATAATAGAAGAGAACATTGAGGGCTTAGTAGGTATCGAGAATAACTCATTCCTTGAGGCACGTAACTTGTTTGATTCGTACCTATCCATCACTATGCCAGACGGACAGCAATTCTCAGTGAATGACCTAAGGGACTTCGATATGTTCCGCATCATGCCAGCGTATGACCGCCGTGTCAATCGTGACATCGCCATCATGGGGTCTACTGGTAAAACCACTTAGGAACTTAAGGATGAGATTTTGGCTCTCAAAGCGAAAGCTGAGGGAGACGGTAAGAAGACTGGCGAg                                              >  1:262811/1‑301 (MQ=255)`  
                                                                                                                                                                                                                                                                 `gagacagggtCCATAAGAGAAGAGAACATTGATGGCTTAGTAGGTATCGAGAATAACTCATTCCTTGATGCACGTAACTTGTTTGATTCGGACCTATCCAGCACTATGCCAGACGGACAGCAATTCTCAGTGAATGACCTAAGGGACTTCGATATGTTCCGCATCATGCCAGCGTATGACCGCCGTGTCAATCGTGACATCGCCAGCATGGGGTCTACTGGTAAAACCACTAAGGAACTTAAGGATGAGATTTTGGCTCTCAAAGCGAAAGCTGAGGGAGACGGTAAGAAGACTGGCGAgg                                             <  1:353582/292‑1 (MQ=255)`  
                                                                                                                                                                                                                                                                       `acagccATAATAGAAGAGAACATTGAGTGCTTAGTAGGTATCGAGAATAACTCATTCCTTGAGGCACGTAACTTGTTTGATTCGGACCTATCCATCACTATGCCAGACGGACAGCAATTCTCAGTGAATGACCTAAGGGACTTCGATATGTTCCGCATCATGCCAGCGTATGACCGCCGTGTCAATCGTGACATCGCCATCATTGGGTCTACTGGTAAAACCACTAAGGAACTTAAGGATGAGATTTTGGCTCTCAAAGCGAAAGCTGAGGGAGACGGTAAGAAGACTGGCGAGGTACATg                                       <  1:642121/297‑1 (MQ=255)`  
                                                                                                                                                                                                                                                                        `gTTCCATAATAGAAGAGAACATTGAGGTCTTAGTAGGTATCGAGAATAACTCATTCCTTGAGGCACGTAACTTGTTTGATTCGGACCTATCCATCACTATGCCAGACGGACAGCAATTCTCAGTGAATGACCTAATGGACTTCGATATGTTCCGCATCATGCCAGCGTATGACCGCCGTGTCAATCGTGACATCGCCATCATGGCGTCTACTGGTAAAACCACTAAGGAACTTAAGGATGAGATTTTGGCTCTCAAAGCGAAAGCTGAGGGAGACGGTAAGAAGACTGGCGAGGTActgtc                                      >  1:34871/1‑297 (MQ=255)`  
                                                                                                                                                                                                                                                                        `gTTCCATAATAGAAGAGAACATTGAGGGCTTAGTAGGTATCGAGAATAACTCATTCCTTGAGGCACGTAACTTGTTTGATTCGGACCTATCCATCACTATGCCAGACGGACAGCAATTCTCAGTGAATGACCTAAGGGACTTCGATATGTTCCGCATCATGCCAGCGTATGACCGCCGTGTCAATCGTGACATCGCCATCATGGGGTCTACTGGTAAAACCACTAAGGAACTTAAGGATGAGATTTTGGCTCTCAAAGCGAAAGCTGAGGgaactgtctcttattcacacctccgagcccc                                      >  1:13862/1‑272 (MQ=255)`  
                                                                                                                                                                                                                                                                         `ttCAATAATAGAAGAGAACATTGAGGGCTTAGTAGTTATCGAGAATAACTCATTCCTTGAGGCACGTAACTTGTTTGATTCGGACCTATCCATCACTATGCCAGACGGACAGCAATTCTCAGTGAATGACCTAAGGGACTTCGATATGTTCCGCATCATGCCAGCGTATGACCGCCGTGTCAATGGTGACATCGCCATCATGGGGTCTACTGGTAAAACCACTAAGGAACTTAAGGATGAGATTTTGGATCTCAAAGCGAAAGCTGAGGGAGACGGTAAGAAGACTGGCGAGGTACATGCt                                     <  1:594653/301‑1 (MQ=255)`  
                                                                                                                                                                                                                                                                           `ccATAATAGAAGAGAACATTGAGGGCTTATTAGCTATCGAGAATAACTCATTCCTTTAGGCACGTAACTTGTTTGATTCGGACCTATCCATCACTATGCCAGACGGACAGCAATTCTCAGTGAATGACCTAAGGGACTTCTATACGTTCCGCATCATGCCAGCGTATGACCGCCGTGTCAATGGTGACATCGCCATCATGGGGTCTACTGGTAAAACCCATAAGGAACTTAAGGATGAGATTTTGGCTCTCAAAGCGAAAGCTGAGGGAGACGGTAAGAAGACTGGCGAGGTACAGGCttt                                   >  1:266784/1‑301 (MQ=255)`  
                                                                                                                                                                                                                                                                           `ccATAATAGAAGAGAACATTGAGGGCTTAGTAGGTATCGAGAATAACTCATTCCTTGAGGCCCGTAACTTGTTTGATTCGGACCTATCCATCACTATTCCAGACGGACAGCAATTCTCAGTGAATGACCTAAGGGACTTCGATATGTTCCGCATCGTGCCAGCGTATGACCGCCGTGTCAATTGTGCCATCGCCATCATGGGGTCTACTGGTAAAACCACTAAGGAACTTAAGGATGAGATTTTGGCTCTCAAAGCGAAAGCTGAGGGAGACGGTCAGACGCCTGGCGAGGTACATGCttt                                   >  1:567173/1‑301 (MQ=255)`  
                                                                                                                                                                                                                                                                              `taataGAAGAGAACATTGAGGGCTTAGTAGGTATCGAGAATAACTCATTCCTTTAGGCACGTAACTTGTTTGATTCGGACCTATCCATCACTATGCCAGACGGACAGCAATTCTCAGTGAATGACCTAAGGGACTTCGATATGTTCTGCATCATGCCAGCGTATGACCTCCGTGTCAATGGTAACATCGCCATCATTGGGTCTACTGGTAAAACCACTAAGGAACTTAAGTATGAGATTTTGGCTCTCAAAGCGAAAGCTGAGGGCGACGGTAAGAAGACTGGCGAGGTACATGCGTTAAt                                >  1:540144/1‑301 (MQ=255)`  
                                                                                                                                                                                                                                                                                `gggctcggagatgtgtataagagacagggTATCGAGAATAACTCATTCCTTGAGGCACGTAACTTGTTTGATTCGGACCTATCCATCACTATGCCAGACGGACAGCAATTCTCAGTGAATGACCTAAGGGACTTCGATATGTTCCGCATCATGCCAGCGTATGACCGCCGTGTCAATCGTGACATCGCCATCATGGGGTCTACTGGTAAAACCACTAAGGAACTTAAGGATGAGATTTTGGCTCTCAAAGCGAAAGCTGAGGGAGACGGTAAGAAGACTGGCGAGGTACATGCTTTAATgg                              <  1:190977/274‑1 (MQ=255)`  
                                                                                                                                                                                                                                                                                `ataGAAGAGAACATTGAGGGCTTAGTAGGTATCGAGAATAACTCATTCCTTGAGGCACGTAACTTGTTTGATTCGGACCTATCCATCACTATGCCAGACGGACAGCAATTCTCAGTGAATGACCTAAGGGACTTCGATATGTTCCGCATCATGCCAGCGTATGACCGCCGTGTCAATCGTGACATCGCCATCATGGGGTCTACTGGTAAAACCACTAAGGAACTTAAGGATGAGATTTTGGCTCTCAAAGCGAAAGCTGATGGAGACGGTAAGAAGACTGGCGAGGTACATGCTTTAATgg                              <  1:85858/301‑1 (MQ=255)`  
                                                                                                                                                                                                                                                                                `ataGAAGAGAACATTGAGGGCTTAGTAGGTATCGAGAATAACTCATTCCTTGAGGCACGTAACTTGTTTGATTCGGACCTATCCATCACTATGCCAGACGGACAGCAATTCTCAGTGAATGACCTAAGGGACTTCGATATGTTCCGCATCATGCCAGCGTATGACCGCCGTGTCAATCGTGACATCGCCATCATGGGGTCTACTGGTAAAACCACTAAGGAACTTAAGGATGAGATTTTGGCTCTCAAAGCGAAAGCTGAGGGAGACGGTAAGAAGACTGGCGAGGTACATGCTTTAATgg                              <  1:794603/301‑1 (MQ=255)`  
                                                                                                                                                                                                                                                                                `ataGAAGAGAACATTGAGGGCTTAGTAGGTATCGAGAATAACTCATTCCTTGAGGCACGTAACTTGTTTGATTCGGACCTATCCATCACTATGCCAGACGGACAGCAATTCTCAGTGAATGACCTAAGGGACTTCGATATGTTCCGCATCATGCCAACGTATGACCGCCGTGTCAATGGTGACATCGCCATCATGGGGTCTACTGGTAAAACCACTAAGGAACTTAAGGATGAGATTTTGGCTCTCAAAGTGAAAGCTGAGTGAGACGGTAAGAAGACTGGCGAGGTACATGCTTTAATgg                              <  1:374472/301‑1 (MQ=255)`  
                                                                                                                                                                                                                                                                                  `agaagaGAACATTGAGGGATTAGTAGTTATCGAGAATAACTCATTCCTTGAGGCACGTAACTTGTTTGATTCGGACCTATCAATCACTATGCCAGACGGACAGCAATTCTCAGTGAATGACCTAAGGGACTTCGATATGTTCCGCATCATGCCAGCGTATGACCGCCGTGTCAATCGTGACATCGCCATCATGGGGTCTACTGGTAAAACCACTAAGGAACTTAAGGATGAAATTTTGGCTCTCAAAGCGAAAGCTGAGGGAGACGGTAAGAAGACTGGCGAGGTACATGCTTTAATGGAt                            <  1:251157/301‑1 (MQ=255)`  
                                                                                                                                                                                                                                                                                       `agaACATTGAGGGCTTAGTAGGTATCGAGAATAACTCATTCCTTGAGGCACGTAACTTGTTTGATTCGGACCTATCCATCACTATGCCAGACGGACAGCAATTCTCAGTGAATGACCTAAGGGACTTCGATATGTTCCGCATCATGCCAGCGTATGACCGCCGTGTCAATCGTGACATCGCCATCATGGGGTCTACTGGTAAAACCACTAAGGAACTTAAGGATGAGATTTTGGCTCTCAAAGCGAAAGCTGAGGGAGACGGTAAGAAGACTGGCGAGGTACATGCTTTAATGGATACCGt                       >  1:511642/1‑301 (MQ=255)`  
                                                                                                                                                                                                                                                                                        `gaACATTGAGGGCTTAGTAGGTATCGAGAATAACTCATTCCTTGAGGCACGTAACTTGTTTGATTCGGACCTATCCATCACTATGCCAGACGGACAGCAATTCTCAGTGAATGACCTAAGGGACTTCGATATGTTCCGCATCATGCCAGCGTATGACCGCTGTGTCAATGGTGACATCGCCATCATGGGGTCTACTGGTAAAACCACTAATGAACTTAAGGATGAGATTTTGGCTCTCAAAGCGAAAGCTGAGGGAGACGGTAAGAAGACTGGCGAGGTACATGCTTTAAtctgtctcttt                      >  1:658635/1‑291 (MQ=255)`  
                                                                                                                                                                                                                                                                                        `gaACATTGAGGGCTTAGTAGGTATCGAGAATAACTCATTCCTTGAGGCACGTAACTTGTTTGATTCGGACCTATCCATCACTATGCCAGACGGACAGCAATTCTCAGTGAATGACCTAAGGGACTTCGATATGTTCCGCATCATGCCAGCGTATGACCGCCGTGTCAATAGTGACATCGCCATCATGGGGTCTACTGGTAAAACAACTAAGGAACTTAAGGATGAGATTTTGGCTCTCAAAGCGAAAGCTGAGGGAGACGGTAAGAAGACTGGCGAGGTACATGCTTTAATGGATACCGtt                      >  1:363873/1‑301 (MQ=255)`  
                                                                                                                                                                                                                                                                                           `cATTGAGTGCTTAGTAGGTATCGAGAATAACTCATTCCTTGATGCACGTAACTTGTTTGATTCGTACCTATCCATCACTATGCCATACGGACAGCAATTCTCAGTGAATGACCTAAGGGACTTCGATATGTTCCGCATCATGCCAGCGTATGACCGCCGTGTCAATAGTGACATCGCCATCATGGGGTCTACTGGTAAAACCACTAAGGAAATTAAGGATGAGATTTTGGCTCTCAAAGCGAAAGCTGAGGGAGACGGTAAGAAGACTGGCGAGGTCAATGCTTTAATTGATACCGTTaca                   >  1:149024/1‑299 (MQ=255)`  
                                                                                                                                                                                                                                                                                            `aTTGAGGGCTTAGTAGGTATCGAGAATAACTCATTCCTTGAGGCACGTAACTTGTTTGATTCGGACCTATCCATCACTATGCCAGACGGACAGCAATTCTCAGTGAATGACCTAAGGGACTTCGATATGTTCCGCATCATGCCAGCGTATGACCGCCGTGTCAATCGTGACATCGCCATCATGGGGTCTACTGGTAAAACCACTAAGGAACTTAAGGATGAGATTTTGGCTCTCAAAGCGAAAGCTGAGGGAGACGGTAAGAAGACTGGCGAGGTACATGCTTTAATGGATACCGTTAAGa                  >  1:666028/1‑301 (MQ=255)`  
                                                                                                                                                                                                                                                                                               `gAGGACTTAGTAGTGATAGAAAATAACTCATTCCTTGGGGCACGTAACTGGTTTGATTCGGACCTAGCCATCACTATGCCAGACGGACAGCAATTCTCAGTGAATGACCTAAGTGACTTCGATATGTTCCGCATCATGCCAGCGTATGACTGCCGTGTCAATGGTGACATCGCCATCATGGGGTCTACTGGTAAAACCACTAAGGAACTTAAGGATGAGATTTTGGCTCTCAAAGCGAAAGCTGAGGGAGACGGTAAGAAGACTGGCGGGGTACATGCTTTAATGGATACCGTTAAGATTc               <  1:727288/301‑1 (MQ=255)`  
                                                                                                                                                                                                                                                                                                 `gggCTTAGTAGGTATCGAGAATAACTCATTCCTTGAGGCACGTAACTTGTTTGATTCGGACCTATCCATCACTATGCCAGACGGACAGCAATTCTCAGTGAATGACCTAAGGGACTTCGATATGTTCCGCATCATGCCAGCGTATGACCGCCGTGTCAATCGTGACATCGCCATCATGGGGTCTACTGGTAAAACCACTAAGGAACTTAAGGATGAGATTTTGGCTCTCAAAGCGAAAGCTGAGGGAGACGGTAAGAAGACTGGCGAGGTACATGCTTTAATGGATACCGTTACGATTCtt             >  1:426730/1‑301 (MQ=255)`  
                                                                                                                                                                                                                                                                                                   `gCTTAGTAGTTATCGAGAATAACTCATTCCTTGAGGCACGTAACTTGTTTGATTCGGACCTATCCATCACTATGCCAGACGGACAGCAATTCTCAGTGAATGACCTAAGGGACTTCGATATGTTCCGCATCATGCCAGCGTATGACCGCCGTGTCAATGGTGACATCGCCATCATGGGGTCTACTGGTAAAACCACTAAGGAACTTAAGGATGAGATTTTGGCTCTCAAAGCGAAAGCTGAGGGAGACGGTAAGAAGACTGGCTAGGTACATGCTTTAATGGATACCGTTAAGATTCTTAc           >  1:255009/1‑301 (MQ=255)`  
                                                                                                                                                                                                                                                                                                   `gCTTAGTAGTTATCGAGAATAACTCATTCCTTGAGGCACGTAACTTGTTTGATTCGGACCTATCCATCACTATGCCAGACGGACAGCAATTCTCAGTGAATGACCTAAGGGACTTCGATATGTTCCGCATCATGCCAGCGTATGACCGCCGTGTCAATGGTGACATCGCCATCATGGGGTCTACTGGTAAAACCACTAAGGAACTTAAGGATGAGATTTTGGCTCTCAAAGCGAAAGCTGAGGGAGACGGTAAGAAGACTGGCGAGGTACATGCTTTAATGGATACCGTTAAGATTCTTAc           >  1:439579/1‑301 (MQ=255)`  
                                                                                                                                                                                                                                                                                                   `gCTTAGTAGGTATCGAGAATAACTCATTCCTTGAGGCACGTAACTTGTTTGATTCGGACCTATCCATCACTATGCCAGACGGACAGCAATTCTCAGTGAATGACCTAAGGGACTTCGATATGTTCCGCATCATGCCAGCGTATGACCGCCTTGTCAATCGTGACATCGCCATCATGGGGTCTACTGGTAAAACCACTAAGGAACTTAAGGATGAGATTTTGGCTCTCAAAGCGAAAGCTGAGGGAGACGGTAAGAAGACTGGCGAGGTACATGCTTTAATGGATACCATTAAGATTCTTAc           <  1:507272/301‑1 (MQ=255)`  
                                                                                                                                                                                                                                                                                                           `ggTATCGAGAATAACTCATTCCTTGAGGCACGTAACTTGTTTGATTCGGACCTATCCATCACTATGCCAGACGGACAGCAATTCTCAGTGAATGACCTAAGGGACTTCGATATGTTCCGTATCATGCCAGCGTATGACCGCAGTGTCAATGGTGACATCGCCATCATGGGGTCTACTGGTAAAACCACTAAGGAACTTAAGGATGAGATTTTGGCTCTCAAAGCGAAAGCTGAGGGAGCCGGTAAGAAGACTGGCGAGGTACATGCTTTAATGGATACCGTTAAGATTCTTAcctgtctct   >  1:714689/1‑293 (MQ=255)`  
                                                                                                                                                                                                                                                                                                            `gTATCTAGAATAACTCATTCCTTGAGGCACGTAACTTGTTTGATTCGGACCTATCCATCACTATGCCAGACGGACAGCAATTCTCAGTGAATGACCTAAGGGACTTCGATATGTTCCGCATCATGCCAGCGTATGACCGCCGTGTCAATTGTGACATCGCCATCATGGGGTCTACTGGTAAAACCACTAAGGAACTTAAGGATGAGATTTTGGCTCTCAAAGCGAAAGCTGAGGGAGACGGTAAGAAGACTGGCGAGGTACATGCTTTAATGGATACCGTTAAGATTCTTACTGGTCGTGc  >  1:700514/1‑301 (MQ=255)`  

                                                                                                                                                                                                                                                                                                            `|`                                                                                                                                                                                                                                                                                                              
`GTCGCCACAAAGGTACTTACGTTCCTCACGTATATGACCGTCATGCCAAGGCGCTGATGATTCAACGCTACGGTGCCGAAGGTTTGCAGGAAGGGATTGCCCGCTCATGGATGAACAGCTACGTCTCCAGACCTGAGGTCAAGGCCAGAGTCGATGAGATGCTTAAGGAATTACACGGGGTGAAGGAAGTAACACCAGAGATGGTAGAGAAGTACGCTATGGATAAGGCTTATGGTATCTCCCACTCAGACCAGTTCACCAACAGTTCCATAATAGAAGAGAACATTGAGGGCTTAGTAGGTATCGAGAATAACTCATTCCTTGAGGCACGTAACTTGTTTGATTCGGACCTATCCATCACTATGCCAGACGGACAGCAATTCTCAGTGAATGACCTAAGGGACTTCGATATGTTCCGCATCATGCCAGCGTATGACCGCCGTGTCAATGGTGACATCGCCATCATGGGGTCTACTGGTAAAACCACTAAGGAACTTAAGGATGAGATTTTGGCTCTCAAAGCGAAAGCTGAGGGAGACGGTAAGAAGACTGGCGAGGTACATGCTTTAATGGATACCGTTAAGATTCTTACTGGTCGTGC  >  NC_001604.1/32570‑33170`

  

Alignment Legend

Aligned base mismatch/match (shaded by quality score): `ATCG/ATCG` `< 3 ≤` `ATCG/ATCG` `< 11 ≤` `ATCG/ATCG` `< 27 ≤` `ATCG/ATCG` `< 37 ≤` `ATCG/ATCG` `< 38 ≤` `ATCG/ATCG`

Unaligned base: `atcg`    Masked matching base: `atcg`    Alignment gap: `‑`    Deleted base: `‑`
